# Editorial: Phosphorylation-dependent peptidyl-prolyl cis/trans isomerase PIN1 - volume II

**DOI:** 10.3389/fcell.2024.1519874

**Published:** 2024-11-07

**Authors:** Futoshi Suizu

**Affiliations:** Laboratory of Pathology, Department of Medical Technology, Kagawa Prefectural University of Health Sciences, Takamatsu, Kagawa, Japan

**Keywords:** PIN1, peptidyl-prolyl cis-trans isomerase, PIN1 inhibitor, PIN1 structure, cancer, viral infection, dementia, tau pathology

In the first volume of our comprehensive exploration into the world of phosphorylation-dependent peptidyl-prolyl cis/trans isomerase PIN1, we established a foundational understanding of this enzyme’s structural and functional dynamics (https://www.frontiersin.org/research-topics/10042/phosphorylation-dependent-peptidyl-prolyl-cistrans-isomerase-pin1). We examined its pivotal role in regulating protein function through its unique ability to catalyze the cis/trans isomerization of proline residues in phosphorylated substrates. This initial volume provided a thorough overview of PIN1’s involvement in various cellular processes, including cell cycle regulation, signal transduction, and apoptosis. Volume II builds upon this foundation, introducing new dimensions and insights into the multifaceted nature of PIN1. The Research Topic of original research articles and reviews represents a significant contribution to our understanding of PIN1 biology and its diverse roles in health and disease.

In this volume, we delve into the multifaceted functions of PIN1 across various physiological and pathological contexts, ranging from cancer to neurodegeneration, vascular disorders, and viral infections. The research presented here sheds light on the molecular mechanisms underlying PIN1-mediated signaling pathways and explores the therapeutic implications of targeting PIN1 in disease intervention.

We begin with original research articles that unveil novel insights into PIN1 biology and its implications for disease pathology and treatment. The development of a contacting transwell co-culture system for the *in vitro* propagation of primary central nervous system lymphoma offers a valuable tool for studying this aggressive malignancy and investigating potential therapeutic strategies (Nishi et al.). Similarly, research elucidating the role of PIN1 in stabilizing NeuroD during the differentiation of mechanoreceptors provides new insights into sensory neuron development and may hold implications for sensory disorders (Zhao et al.).

Furthermore, original research articles highlight the therapeutic potential of PIN1 modulation in cancer and other diseases. KPT6566, a promising inhibitor of PIN1, demonstrates efficacy in inducing apoptotic cell death and suppressing the tumorigenicity of testicular germ cell tumors, suggesting its potential as a targeted therapy for this malignancy (Sun et al.). Similarly, juglone and KPT6566 show promise in suppressing the tumorigenic potential of CD44^+^CD133^+^ tumor-initiating cells in colorectal cancer, offering new avenues for therapeutic intervention (Sun et al.).

In addition to original research, our volume features reviews and mini reviews that provide comprehensive overviews of PIN1 biology and its roles in disease. The review on the PIN1-cis P-tau axis explores its significance in the development and treatment of vascular contribution to cognitive impairment and dementia, as well as preeclampsia, shedding light on the complex interplay between PIN1 and tau pathology in these conditions (Qiu et al.). Another review delves into the role of PIN1 as a master cancer regulator, highlighting its diverse functions in cancer development and treatment and underscoring its potential as a therapeutic target (Stewart et al.).

Moreover, a mini review explores the roles of PIN1 in viral propagation, emphasizing its importance in modulating viral protein function and interactions with host factors (Kanna et al.). Additionally, a review examines the structural and functional convergence and divergence of PIN1, providing insights into its diverse functions across different cellular contexts and diseases (Lee et al.).

Collectively, the articles in this volume contribute to our growing understanding of the intricate roles of PIN1 in cellular physiology and pathology. As we navigate through the complexities of PIN1 biology, it becomes increasingly clear that a multidisciplinary approach is essential to fully comprehend its diverse roles in health and disease. By integrating knowledge from fields such as biochemistry, structural biology, cell biology, and medicine, we can gain a more comprehensive understanding of PIN1 function and its implications for human health. We extend our sincere appreciation to the authors for their outstanding contributions and to the readers for their interest in this Research Topic. Together, let us continue to unravel the complexities of phosphorylation-dependent peptidyl-prolyl cis/trans isomerase PIN1 and explore its therapeutic potential for improving human health and wellbeing.

